# Does indocyanine green fluorescence angiography impact the intraoperative choice of procedure in free vascularized medial femoral condyle grafting for scaphoid nonunions?

**DOI:** 10.3389/fsurg.2022.962450

**Published:** 2022-09-02

**Authors:** Markus Mulica, Raymund E. Horch, Andreas Arkudas, Aijia Cai, Wibke Müller-Seubert, Theresa Hauck, Ingo Ludolph

**Affiliations:** Department of Plastic and Hand Surgery, Laboratory for Tissue Engineering and Regenerative Medicine, University Hospital Erlangen, Friedrich-Alexander University Erlangen-Nuernberg (FAU), Erlangen, Germany

**Keywords:** scaphoid nonunion, free vascularized bone graft, medial femoral condyle bone graft, indocyanine green angiography, union rate

## Abstract

**Background:**

Free vascularized medial femoral condyle (MFC) bone grafts can lead to increased vascularity of the proximal pole and restore scaphoid architecture in scaphoid nonunions. The intraoperative perfusion assessment of the bone graft is challenging because the conventional clinical examination is difficult. Indocyanine green (ICG) angiography has previously been shown to provide a real-time intraoperative evaluation of soft tissue perfusion in reconstructive surgery. The present study investigated the utility of ICG angiography in patients treated with a free medial femoral condyle graft for scaphoid nonunions.

**Methods:**

We performed a retrospective analysis of patients with scaphoid nonunions, in which ICG angiography was used intraoperatively for perfusion assessment. The medical records, radiographs, intraoperative imaging, and operative reports of all patients were reviewed. Intraoperative ICG dye was administered intravenously, and laser angiography was performed to assess bone perfusion. The scaphoid union was examined using postoperative CT scans.

**Results:**

Two patients had documented osteonecrosis of the proximal pole at the time of surgery. Four patients received a nonvascularized prior bone graft procedure, and a prior spongiosa graft procedure was performed in one patient. The mean time from injury to the MFC bone graft surgery was 52.7 months, and the mean time from prior failed surgery was 10.4 months. Perfusion of the vascular pedicle of the MFC and the periosteum could be detected in all patients. In two patients, even perfusion of the cancellous bone could be demonstrated by ICG angiography. Following transplantation of the bone graft, patency of the vascular anastomosis and perfusion of the periost were confirmed by ICG angiography in the assessed cases. No additional surgery regarding a salvage procedure for a scaphoid nonunion advanced collapse was necessary for the further course.

**Conclusion:**

ICG-angiography has shown to be a promising tool in the treatment of scaphoid nonunion with medial femoral condyle bone grafts. It enables intraoperative decision making by assessment of the microvascular blood supply of the periosteum and the vascular pedicle of the MFC bone graft. Further studies need to evaluate the impact on union rates in a long-term follow-up.

## Introduction

Scaphoid fractures account for up to 70% of all carpal fractures. Approximately 5%–15% of overall acute scaphoid fractures develop a scaphoid nonunion (1). Risk factors that can induce a nonunion are multifactorial: delayed cast immobilization, fracture displacement, proximal location of the fracture, and associated carpal instability, among others (2–4). Until today, it is challenging to achieve bony union of a scaphoid nonunion that is associated with osteonecrosis of the proximal pole. Particularly challenging are those in which a scaphoid nonunion is associated with a humpback deformity and a carpal collapse stage I (5, 6). In patients with nonunion of the scaphoid combined with osteonecrosis, nonvascularized bone grafting results in union rates of only 47% based on a recent meta-analysis of the literature. In these patients, vascular bone grafting results in more reliable and consistent union rates of up to 88%, even in the subset of patients with both scaphoid collapse and proximal pole necrosis (2). Vascularized bone grafting offers the potential benefit of increased osteochondral viability, which enhances the revascularization of partially necrotic bone and promotes bony union. This procedure may be of particular utility in patients with proximal pole necrosis and previously failed surgeries including internal fixation or nonvascularized bone grafting accompanied by a long duration of nonunion. Various techniques of vascular interposition grafts, pedicled and free, have been reported in the literature for nonunion of scaphoid fractures. Numerous authors described the medial femoral condyle as a valuable source of a vascularized bone graft for successful treatment of nonunion of the humerus, clavicula, and tibia (7). The therapeutical use of a free vascularized graft from the medial femoral condyle (MFC) for scaphoid nonunion was first described by Doi et al. (8). The MFC graft offers multiple advantages, including a robust vascular supply, excellent bone quality regarding bone density, and the possibility of reshaping an individually customized graft for structural support of the scaphoid (9). Reliable perfusion of the medial femoral bone graft is decisive for revascularization of the proximal necrotic scaphoid pol and successful restoration of the scaphoid. Despite the increasing interest in the use of free vascularized bone grafts in the surgical treatment of scaphoid nonunions, an appropriate intraoperative imaging technique regarding vascularisation of the free bone graft has not been studied yet. This study aimed to provide insights into the application of indocyanine green (ICG) as a well-known intraoperative tissue perfusion analysis tool in patients treated with a free vascularized medial femoral condyle bone graft.

## Materials and methods

We performed a retrospective review of patients with a scaphoid nonunion that were treated with a free vascularized medial femoral condyle bone graft and in whom ICG angiography was used to analyze its value for perfusion assessment of the corticocancellous graft. Medical records, radiographs, intraoperative imaging, and operative reports were reviewed. In all patients, regardless whether first-line treatment or revision surgery, a free vascularized MFC bone graft was performed if they were deemed to have sufficient proximal pole bone stock to permit at least Kirschner wire stabilization and if no arthritis beyond SNAC (scaphoid nonunion advanced collapse) Stage 1 on preoperative radiographic imaging and intraoperative inspection of the articular surfaces was present.

### Preoperative evaluation

Preoperatively, all patients underwent a physical examination. Furthermore, we obtained standard radiographic imaging along with anteroposterior and lateral views of the wrist. To assess the fracture orientation and proximal pole size of the scaphoid, additional computed tomography (CT) was conducted. Evidence for avascular necrosis of the proximal pol was obtained by preoperative magnetic resonance imaging (MRI), which was conducted in all cases. Written informed consent was obtained from all patients, and the study was conducted in accordance with the Declaration of Helsinki. The protocol was approved by the institutional review board (registration number 85_13 B).

### Surgical technique

**Figure 1:**
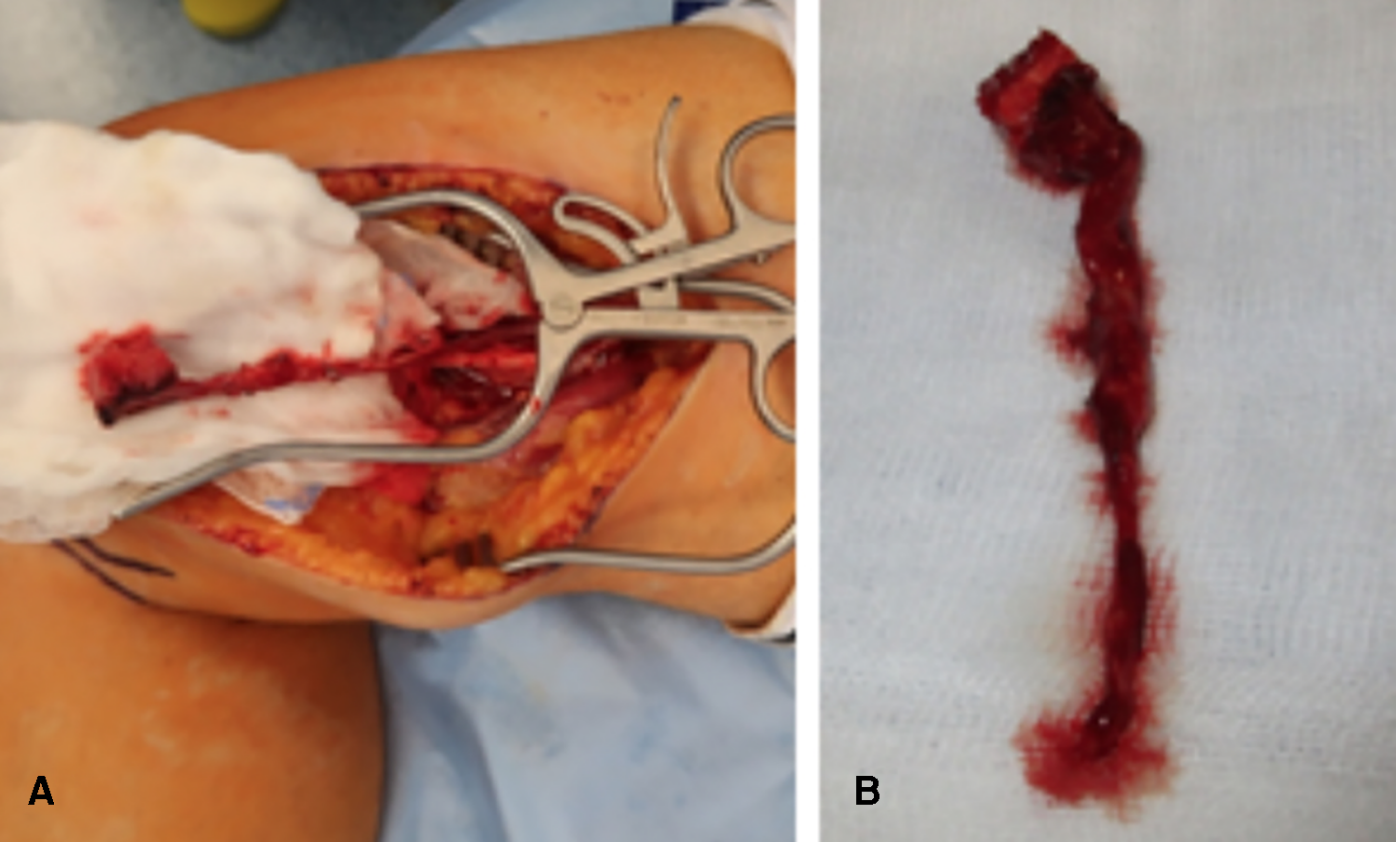
(**A**) Intraoperative view showing the harvesting of a medial femoral condyle. (**B**) Intraoperative view showing the harvested bone graft with a long pedicle.

The surgical technique has been previously described (10, 11). In all patients, a single palmar approach was used. Once the scaphoid is exposed, the nonunion site is examined, and the vascularity of the proximal pole is evaluated. Sclerotic bone structure associated with the absence of punctate bleeding when the tourniquet is released is considered the ultimate evidence of osteonecrosis (12). If necessary, the screw that has been placed in a prior surgery in the scaphoid is removed. Necrotic bone and interposed fibrous tissue are then resected. Subsequently, the opposing edges of the nonunion site are prepared for insertion of the vascularized bone graft. The required graft size can now be measured three-dimensionally according to the bony defect of the scaphoid. The ipsilateral leg in six cases and the contralateral leg in one case were chosen for the MFC bone graft under tourniquet control. Proper positioning of the graft is respected to include visible nutrient periosteal vessels based on all our presented cases on the articular branch of the descending geniculate artery and vein. The edges of the graft are outlined on the periosteum. A straight and curved osteotome is used to divide the graft from the cortex. Care is taken to avoid separating the periosteum from the corticocancellous portion of the graft (Figure 1). After elevation of the graft but before the division of the vascular pedicle, the tourniquet is released and ICG angiography is performed in a standard mode to assess the patency and continuity of the descending geniculate artery and vein as well as the perfusion of the bone graft. In the case of appropriate perfusion, the MFC bone is transplanted to the scaphoid. Prior to inserting the graft to the bony defect of the scaphoid, an additional cancellous bone from the femur can be used, if necessary, to fill a screw hole in the scaphoid. Following the three-dimensional shaping of the MFC bone graft, it is placed as a wedge graft, which fills and slightly expands the scaphoid defect. Ideally, a cannulated scaphoid screw is used to stabilize the inserted graft in a proper alignment. In cases of a very small residual proximal scaphoid pole, Kirschner wires are used. Using microscope magnification, the descending genicular artery is anastomosed end-to-side to the radial artery to avoid compromised circulation of the hand. The concomitant vein is anastomosed end-to-end with a radial vena comitans using either a hand suture or a microvascular hand coupler (Synovis Micro Companies Alliance, Inc., Birmingham, AL, USA). Additionally, another perfusion measurement using ICG angiography can be performed after anastomosis to assess the patency of the pedicle vessels. The defect created at the donor site in the medial femoral condyle can be filled with a bioactive glass (Bonealive) before final wound closure.

### Indocyanine green angiography

As a method of perfusion analysis of the medial femoral condyle bone graft, we used ICG angiography (SPY Elite, Stryker Corporation, Kalamazoo, Michigan, USA). Exclusion criteria were solely based on contraindications for the application of indocyanine green (e.g., iodine allergy, autonomous adenoma of the thyroid gland, hyperthyroidism, or due to refusal by the patient). In all free vascularized MFC bone grafts, ICG angiography was performed in a standard mode using a 12.5 mg (ICG 2.5 mg/ml in water for injections) bolus of ICG dye followed by a saline flush through a venous line after harvesting of the bone graft but before vascular pedicle division. If perfusion was also assessed after anastomosis, the same dosing of the dye was used. ICG binds to plasma proteins confined within the intravascular compartment and thus remains within the blood circulation (13, 14). A further unique property of ICG dye is the short plasma half-life of 150-180 s due to its hepatic metabolism, allowing repeated injections. Upon injection, the area of interest is excited by a near-infrared (NIR) laser. The ICG dye has an absorption maximum at 805 nm and an emission maximum at 835 nm. The fluorescence intensity serves as an indicator for ICG dye uptake and the clearance within the tissue and is displayed in real time on a video monitor. Depending on the extent of ICG dye uptake, the region of interest appears bright if well perfused, whereas regions of mal-perfusion are depicted darkly. In this study, the analysis time was set at a minimum of 120 s. In a standard mode, the captured image is illustrated as a grayscale system.

### Postoperative rehabilitation

Postoperatively, the wrist was immobilized in a palmar long-arm thumb spica splint. After 2 weeks, the palmar long-arm thumb spica splint was changed to a palmar long-arm thumb spica cast, recommended for additional 10 weeks. Postoperative radiographic imaging was conducted after 4 and 8 weeks. In the case of the use of Kirschner wires, they were removed after 12 weeks. For determination of a scaphoid union, a routine CT scan was conducted immediately after removal of Kirschner wires or removal of the cast and after 6 months postoperatively.

## Results

ICG angiography was used for the perfusion analysis of a free vascularized medial femoral condyle graft in seven patients. Two of the included patients had scaphoid nonunions with osteonecrosis of the proximal pole diagnosed by preoperative MRI. Five patients presented with a persistent scaphoid nonunion despite previous surgical intervention with nonvascularized bone grafts (*n* = 4) or spongiosa grafts (*n* = 1). The initial fixation of the scaphoid fracture was done with a cannulated bone screw in four patients and with Kirschner wires in one patient. The patient characteristics and the types of previous surgical intervention are given in Table 1. The average age was 28.4 years (range: 18–55 years) at the time of the MFC bone graft procedure. The average maximal length of the proximal pole of the scaphoid, which was measured in the preoperative coronal CT scan was 8.2 mm (range: 5.4–11.3 mm). The mean time from injury to the MFC bone graft surgery was 52.7 months (range: 12–240 months), and the mean time from prior failed surgery was 10.4 months (range: 5–23 months). The intraoperative details regarding arterial anastomosis, venous anastomosis, graft fixation, and CT follow-up are given in Table 2. For all patients, follow-up with CT scan was at least 3 months (*n* = 7) after scaphoid reconstruction. The mean follow-up time with CT scan was 9.9 months (range: 6–22 months).

**Table 1:** Patients’ characteristics and the types of previous surgical intervention.

Case	Age	Sex	Previous bone graft	Time from injury to MFC graft (months)	Time from previous surgery to MFC graft (months)	Preoperative maximal proximal pole length (mm)	Preoperative proximal pole viability in MR imaging
1	33	M	Bone graft and screw	26	23	9.3	Yes
2	18	M	None	12	n/a	8.7	No
3	14	M	Bone graft and screw	16	11	5.4	Yes
4	24	M	None	38	n/a	7.2	No
5	55	M	Spongiosa and screw	13	6	8.8	Yes
6	23	M	Bone graft and screw	24	7	6.7	Yes
7	37	M	Bone graft and K-wires	240	5	11.3	Yes

MFC, medial femoral condyle; MR imaging, magnetic resonance imaging; n/a, not available.

**Table 2:** Intraoperative details of the patients.

Case	MFC fixation	Arterial anastomosis	Venous anastomosis	Max. CT follow-up (months)
1	2×K-wires	Hand anastomosis end-to-side	1.5 mm venous coupler end-to-end	11
2	2×K-wires	Hand anastomosis end-to-side	Hand anastomosis end-to-end	6
3	2×K-wires	Hand anastomosis end-to-side	1.5 mm venous coupler end-to-end	12
4	Cannulated screw	Hand anastomosis end-to-side	Hand anastomosis end-to-end	22
5	Cannulated screw	Hand anastomosis end-to-side	Hand anastomosis end-to-end	6
6	Cannulated screw	Hand anastomosis end-to-side	1.5 mm venous coupler end-to-end	6
7	2 K-wires	Hand anastomosis end-to-side	2.0 mm venous coupler end-to-end	6

**Figure 2:**
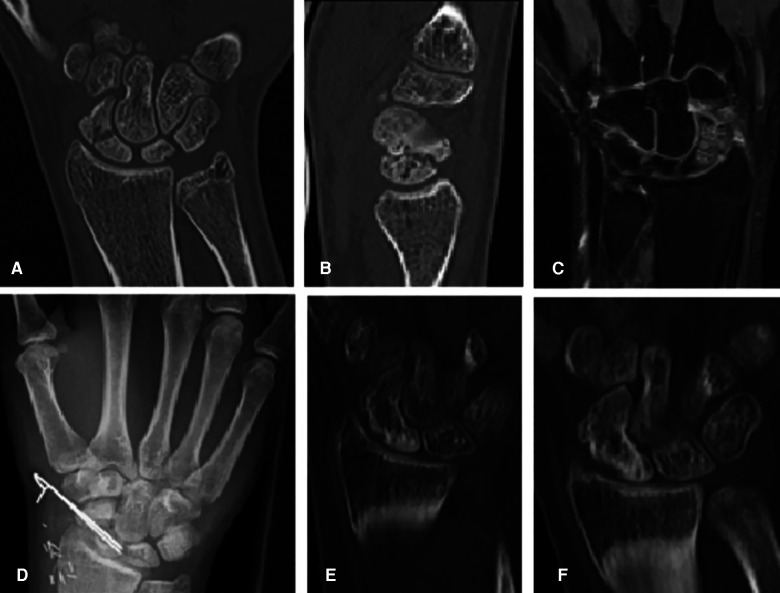
(**A**) Coronal CT scan showing the mid-waist of an 18-year-old male patient with a scaphoid nonunion 1 year after sustaining a scaphoid fracture, which initially remained undetected. (**B**) Sagittal CT scan. (**C**) Coronal gadolinium-enhanced MR imaging scan showing avascular necrosis of the proximal pole. (**D**) Dorsopalmar radiograph postoperative after implantation and Kirschner wire fixation of a vascularized bone graft from the medial femoral condyle. (**E**) Coronal CT scan 3 months postoperative showing complete healing. (**F**) Coronal CT scan 6 months postoperative showing complete healing. MFC, medial femoral condyle; K-wire, Kirschner wire; MR imaging, magnetic resonance imaging.

**Figure 3:**
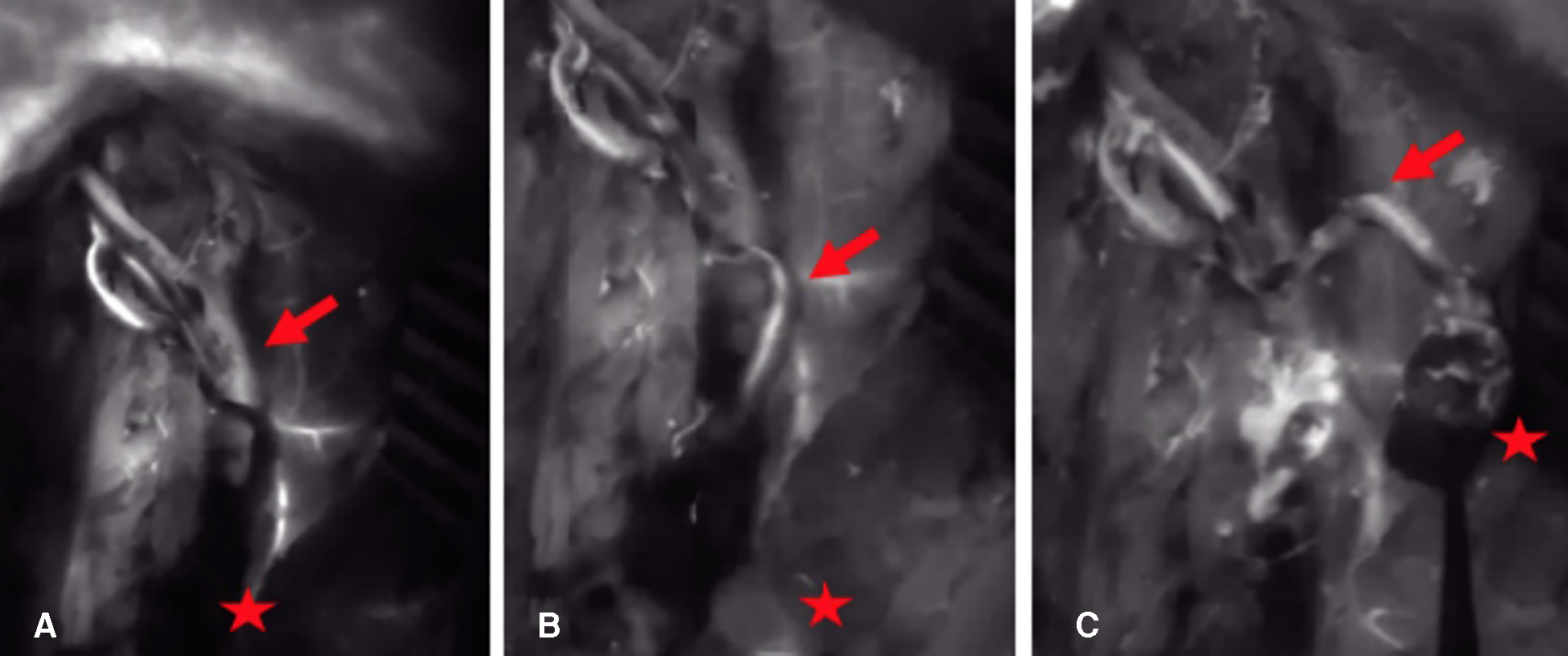
(**A**) 15 s after application of ICG dye: there is no NIR fluorescence signal in the arterial vascular pedicle and the periosteum of the bone graft (arrow = arterial pedicle, star = corticocancellous MFC graft). (**B**) 30 s after application of ICG dye: the parental descending geniculate artery shows a fluorescence signal (arrow = arterial pedicle, star = corticocancellous MFC graft). (**C**) 45 s after application of ICG dye: the parental descending geniculate artery and the periosteum show a fluorescence signal (arrow = arterial pedicle, star = corticocancellous MFC graft). ICG, indocyanine green; NIR, near-infrared; MFC, medial femoral condyle.

**Figure 4:**
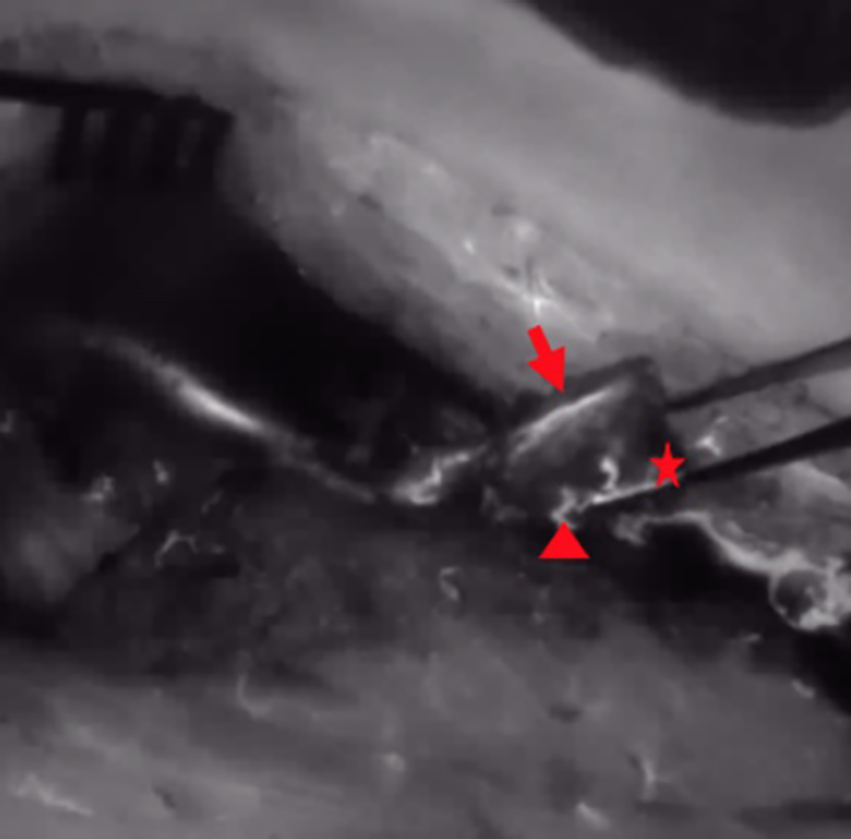
After ICG dye injection, NIR perfusion of the periosteum and the osteotomy site and cut edge (arrow = periosteum, star = osteotomy site, triangle = nutrient vessel at the osteotomy site); ICG, indocyanine green; NIR, near-infrared.

**Figure 5:**
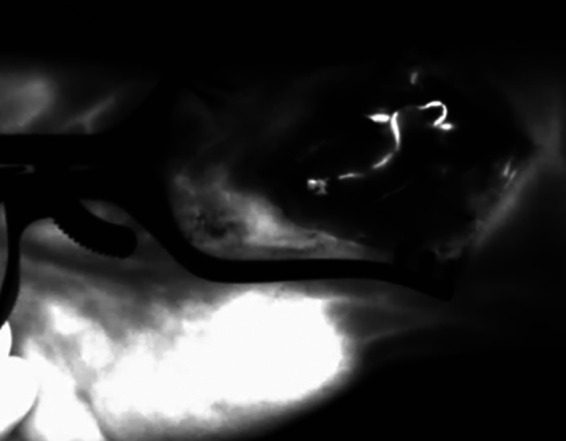
After end-to-side anastomosis of the pedicle artery to the radial artery and ICG dye application. The ICG angiography signal indicates patency of the arterial vascular pedicle of the bone graft; ICG, indocyanine green; NIR, near-infrared.

All arterial anastomoses were performed by hand suture with interrupted nylon sutures end to side to the radial artery. The venous anastomoses were performed end to end to a concomitant vein. In four cases, a venous coupler device (Synovis Micro Companies Alliance, Inc., Birmingham, AL, USA) was used for anastomosis, whereas in three cases, the anastomosis was hand-sewn with interrupted nylon sutures. In four cases, Kirschner wires were used for fixation of the graft, which were removed in all cases after 3 months (Figure 2). In three patients, graft fixation was done with a cannulated bone screw, which was removed in two patients during the follow-up period. In both cases, removal of the screw was performed due to the protrusion of the cannulated screw. 12.5 mg of ICG dye was initially injected through a venous line after harvesting the bone graft but before vascular pedicle division. Each medial femoral bone graft was elevated based on the articular branch of the descending geniculate artery and vein. In all patients, perfusion of the vascular pedicle and perfusion of the periosteum were observed (Figure 3). In two patients, nutrient vessels could also be detected in the cancellous bone of the graft by ICG angiography; hence, additional perfusion of the bone graft was observed (Figure 4) (Supplementary Video S1). In two patients, perfusion assessment using 12.5 mg of ICG dye was also performed after anastomosis, illustrating the patency of the pedicle and perfusion of the overlying periosteum of the MFC graft (Figure 5).

Due to the complex anatomical findings for scaphoid reconstruction in all patients, bony union of the scaphoid was assessed using computed tomography (CT). In the CT scan assessment 3 months postoperatively, the union was achieved in two patients and the partial union was achieved in two patients. In the CT scan 6 months postoperatively, the complete union was achieved in two patients and the partial union was achieved in three patients. Six patients underwent subsequent procedures, four of which underwent planned Kirschner wire removal and two underwent screw removal. No additional surgery regarding a salvage procedure for SNAC (scaphoid nonunion advanced collapse) was performed during the follow-up in our institution. No major donor site morbidity was observed. The only discomfort that patients noticed in the area of the medial femoral condyle included postoperative swelling and hypesthesia in the area around the scar. All of the symptoms disappeared within 3 months or less.

## Discussion

Surgical treatment of scaphoid nonunions is a challenging problem for the attending hand surgeon, especially in the subset of those who are associated with a combination of osteonecrosis and a beginning carpal collapse and for which prior revision surgery had failed. All of the investigated patients in this study presented with complex anatomical findings for scaphoid reconstruction, either due to prior failed nonvascularized bone grafting or osteonecrosis of the proximal pole. In patients with poor proximal pole vascularity, nonvascularized bone grafting results in high nonunion rates. A persistent nonunion of the scaphoid results inevitably in disturbed carpal kinematics and will end up in a carpal collapse over time. When the carpal collapse is accompanied by pain and a failure of conservative treatment, salvage surgeries such as scaphoid excision and four-corner fusion or proximal row corpectomy are required (15).

To prevent this scenario and to achieve union in scaphoids with osteonecrosis, it is necessary to achieve revascularization of the proximal pole, restore the scaphoid architecture, and address carpal stability. In such cases, application of a vascularized bone graft, as the free vascularized medial femoral condyle, is required (16). Pedicled vascular bone grafts, as first described by Zaidemberg for the distal dorsoradial radius, are operative alternatives in selected cases (17). In comparison to a pedicled bone graft from the distal radius, a free vascularized bone graft from the medial femoral condyle offers a higher range of motion of the bone graft and the possibility of harvesting an osteocartilaginous graft as well as high bone quality (6). The autonomous circulation and the influx of viable osteocytes supplied by the vascular anastomosis may promote the union process in the recipient bone. Furthermore, used as an interposition graft, it can add 8–10 mm length to address scaphoid foreshortening and restore the carpal kinematics by providing structural support to the bony defect (18). Adequate perfusion and a patent anastomosis of the bone graft are essential for a successful reconstruction. However, perfusion monitoring of vascularized bone grafts is explicitly challenging. Regular methods of monitoring used in soft tissue flaps as assessing the capillary refill or tissue color are absent in bone grafts. Attempts of a hand-held Doppler for identifying the vascular pedicle of the bone graft are limited due to the interference from neighboring vessels (19). Utilization of ICG fluorescence angiography is a well-standardized laser-driven technology for real-time perfusion analysis of tissue perfusion in various fields to differentiate between viable and nonviable tissue (20, 21). In reconstructive surgery, ICG has been mostly investigated in the perioperative assessment of soft tissue perfusion, e.g., in autologous and alloplastic breast reconstruction surgery (22). However, little is known about the value of ICG angiography in free vascularized bone grafts (23). This study focused on the perfusion assessment and potentially intraoperative decision-making subsequent to intraoperative application of ICG after harvesting and anastomosis of the free medial femoral condyle in patients with scaphoid nonunion.

Yoshimatsu et al. illustrated the feasibility of bone perfusion in cadaveric studies using ICG. The descending genicular artery was dissected and cannulated in four cadavers (24). After injection of 5 ml of ICG solution into the descending genicular artery, the dye has not only been observed in the periosteum but also in the endosteal region using fluorescence angiography. Yoshimatsu et al. attributed this to small perforators penetrating the periosteum and supplying blood to the endosteal part of the bone. The concept is based on a dual osseous angioarchitecture blood supply theory, with direct communication between the periosteal and endosteal network sustaining perfusion even with the loss of the primary nutrient artery of the endosteal portion, which was first postulated by Oni et al. in 1980 (25). The limitations of cadaveric studies are undeniable. One of them could be the oversensitivity of this method due to the isolated injection of ICG into the descending genicular artery, which may distort and overestimate the perfusion assessment of the harvested bone graft. Intraoperative ICG is injected through the venous line, which results in a lower concentration in the region of interest compared to a direct injection of the ICG dye into the parental nutrient vessel, which is also associated with likely different intravascular pressure levels and blood flow characteristics. To the best of our knowledge, no *in vivo* study concerning perfusion analysis of small vascularized free bone grafts exists. In our study, we could observe ICG dye uptake of the vascular pedicle in all patients proving vessel patency and perfusion of at least the periosteum of the bone graft, even in very small-bored vessels after harvesting of the bone graft. Moreover, subsequent perfusion of the periosteum covering the bone graft could be detected. Using ICG angiography, the endosteal network of the medial femoral bone graft was also highlighted if the arterioles running inside the cancellous bone could be displayed at the cut end of the bone graft. If the nutrient arteries were not displayed at the cut end of the bone graft, there was no highlighted perfusion pattern of the cancellous bone portion within a minimum of 120 s. Even though in the majority only perfusion of the overlying periosteum was depicted with ICG angiography, this does not exclude perfusion of cancellous bone in the graft itself. The absence of the ICG dye uptake in the endosteal portion of the bone graft might also be attributed to the technical limitations of the measuring device. In theory, one can assume that if periosteal perfusion is given, the cancellous portion of the bone graft should also be perfused. On the other hand, especially in small bone grafts, there is no proof of a circulation of the cancellous bone by defined vessels originating from the overlying periosteum. In such cases, bone nutrition is rather given by diffusion and not by vascularization of the bone graft itself.

Despite the technical limitations, ICG angiography is a suitable device for perfusion assessment of the vascularized MFC bone graft. Because of the usually small area of the bone graft, defining areas of well-perfused or mal-perfused zones is technically not feasible as known from pedicled or free flaps (26, 27). Furthermore, due to the particular vascularization of small bone grafts, judging the venous phase of the grafts is challenging and was not possible in the presented cases. However, until today, analyzing the venous perfusion pattern is not as well understood as arterial perfusion using ICG angiography in other fields of plastic surgery (13).

Another advantage of ICG angiography is the possibility of assessing perfusion at the level of the vascular pedicle. After harvesting the bone graft, assessing the pedicle with ICG angiography enables the surgeon to identify the origin of a potential vascular compromise of the blood supply and allows the surgeon to intervene, if necessary, before dissecting the graft. Moreover, assessment of the vascular pedicle with ICG angiography after microvascular anastomosis allows the surgeon to detect arterial and venous vascular abnormalities, including spasms, thrombosis, or technical failure of the anastomosis. This additional information can potentially change the intraoperative strategy and therefore enables immediate decision-making.

ICG angiography is independent of the investigator and reliably reproducible (28). The short half-time of the ICG dye enables a measurement after harvesting and after anastomosis of the bone graft. However, ICG angiography has limitations. It represents an invasive procedure with an intravenous application of the dye (29). One has to consider that repeatedly conducted measurements in short intervals develop an increasing background fluorescence, which can lead to a disturbed perfusion assessment. Due to the small size of the bone graft and the small study population, valid quantitative analysis of intraoperative perfusion parameters was not possible in the presented study.

In cases of medial femoral bone grafts, in which a clinical perfusion assessment is difficult, ICG angiography has shown to be a promising tool for intraoperative measurement and assessment of the microvascular blood supply of the periosteum and even of the cancellous bone if perfusion *via* the overlying periosteum is effectively given by vessels. It provides the surgeon with real-time feedback during surgery regarding the patency of the vascular pedicle and allows for intervention if vascular problems are present. It has the potential to enhance the safety of the procedure and provide new insights in the physiological perfusion of especially small bone grafts. However, further studies are required to evaluate a possible influence on the long-term outcomes regarding union rates of the scaphoid in a larger population. Moreover, future studies could investigate the anatomical perfusion pattern of the whole medial femoral condyle. Subsequently, zones of different perfusion levels of the medial femoral condyle could be defined, which could outline the ideal positioning of the MFC bone graft osteotomy sites.

## Data Availability

The original contributions presented in the study are included in the article/**Supplementary Material**, further inquiries can be directed to the corresponding author.
