# Corrigendum: Does indocyanine green fluorescence angiography impact the intraoperative choice of procedure in free vascularized medial femoral condyle grafting for scaphoid non-unions?

**DOI:** 10.3389/fsurg.2022.1101481

**Published:** 2023-01-25

**Authors:** Markus Mulica, Raymund E. Horch, Andreas Arkudas, Aijia Cai, Wibke Müller-Seubert, Theresa Hauck, Ingo Ludolph

**Affiliations:** Department of Plastic and Hand Surgery, Laboratory for Tissue Engineering and Regenerative Medicine, University Hospital Erlangen, Friedrich Alexander University Erlangen-Nürnberg FAU

**Keywords:** scaphoid non-union, free vascularized bone graft, medial femoral condyle bone graft, indocyanine green angiography, union rate

A Corrigendum on Does indocyanine green fluorescence angiography impact the intraoperative choice of procedure in free vascularized medial femoral condyle grafting for scaphoid nonunions? By Markus M, Raymund H, Andreas A, Aijia C, Wibke M-S, Theresa H and Ingo L. (2022) Front. Surg. 9:962450. doi: 10.3389/fsurg.2022.962450

**Incorrect Author Name**

In the published article, the authors name were written as Mulica Markus, Horch Raymund, Arkudas Andreas, Cai Aijia, Müller-Seubert Wibke, Hauck Theresa and Ludolph Ingo

The correct name order is (first name and surname).

Markus Mulica, Raymund E. Horch, Andreas Arkudas, Aijia Cai, Wibke Müller-Seubert, Theresa Hauck, Ingo Ludolph

**Error in Figure Legend**

In the published article, there was an error in the legend for Figure 5 as published.

**Figure 5:**
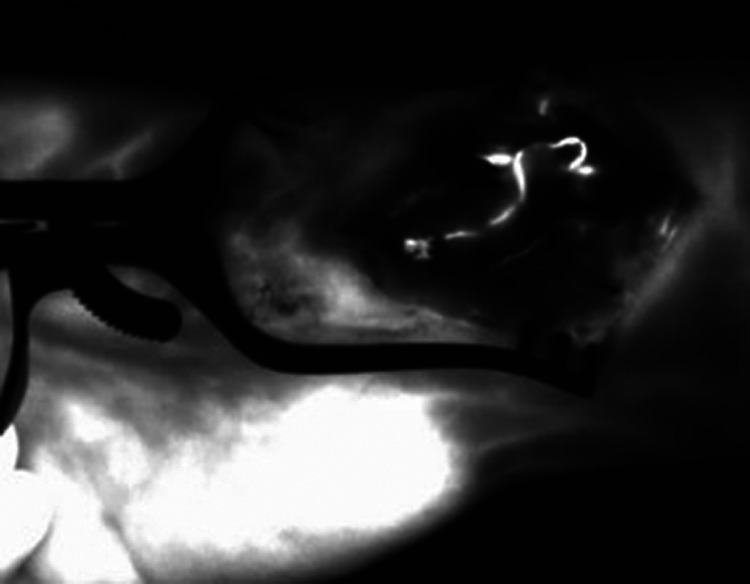
After end-to-side anastomosis of the pedicle artery to the radialis artery and ICG dye application. The ICG angiography signal indicates patency of the arterial vascular pedicle of the bone graft; ICG, indocyanine green; NIR, near-infrared.

The corrected legend appears below.

After end-to-side anastomosis of the pedicle artery to the radial artery and ICG dye application. The ICG angiography signal indicates patency of the arterial vascular pedicle of the bone graft; ICG, indocyanine green; NIR, near-infrared.

**Text Correction**

Below the sections of the article are listed and underlined to which a change was made:

A correction has been made to the following section:

**Surgical technique:**

This sentence previously stated:

The ipsilateral leg in six cases and the contralateral leg in one case were chosen for the MFC bone graft under tourniquet control (Figure 1).

The corrected sentence appears below:

The ipsilateral leg in six cases and the contralateral leg in one case were chosen for the MFC bone graft under tourniquet control.

**Surgical technique:**

This sentence previously stated:

A straight and curved osteotome is used to divide the graft from the cortex. Care is taken to avoid separating the periosteum from the corticocancellous portion of the graft.

The corrected sentence appears below:

A straight and curved osteotome is used to divide the graft from the cortex. Care is taken to avoid separating the periosteum from the corticocancellous portion of the graft (Figure 1).

**Indocyanine green angiography:**

This sentence previously stated:

“A further unique property of ICG dye is the short plasma half-life of 150–1,80 s due to its hepatic metabolism, allowing repeated injections.”

The corrected sentence appears below:

“A further unique property of ICG dye is the short plasma half-life of 150–180 s due to its hepatic metabolism, allowing repeated injections.”

**Indocyanine green angiography:**

This sentence previously stated:

“After the application of SPY Elite, the ICG dye has an absorption maximum at 805 nm and an emission maximum at 835 nm.”

The corrected sentence appears below:

“The ICG dye has an absorption maximum at 805 nm and an emission maximum at 835 nm.”

**Postoperative rehabilitation**

This sentence previously stated:

“After 2 weeks, the palmar long-arm thumb spica splint was changed to a palmar long-arm thumb spica cast, and if swelling decreased to a tolerable level, it was recommended for additional 10 weeks.”

The corrected sentence appears below:

“After 2 weeks, the palmar long-arm thumb spica splint was changed to a palmar long-arm thumb spica cast, recommended for additional 10 weeks.”

**Results:**

This sentence previously stated:

The mean follow-up time with CT scan was 9.9 months (range: 6–22 months) (Figure 2).

The corrected sentence appears below:

The mean follow-up time with CT scan was 9.9 months (range: 6–22 months).

**Results:**

This sentence previously stated:

“In four cases, Kirschner wires were used for fixation of the graft, which were removed in all cases after 3 months.”

The corrected sentence appears below:

“In four cases, Kirschner wires were used for fixation of the graft, which were removed in all cases after 3 months (Figure 2).”

**Results:**

This sentence previously stated:

“About 12.5 mg of ICG dye was initially injected through a venous line after harvesting the bone graft but before vascular pedicle division.”

The corrected sentence appears below:

“12.5 mg of ICG dye was initially injected through a venous line after harvesting the bone graft but before vascular pedicle division.”

**Results:**

This sentence previously stated:

“Six patients underwent subsequent procedures, four of which underwent planned Kirchner wire removal and two underwent screw removal.”

The corrected sentence appears below:

“Six patients underwent subsequent procedures, four of which underwent planned Kirschner wire removal and two underwent screw removal.”

The authors apologize for this error and state that this does not change the scientific conclusions of the article in any way. The original article has been updated.

